# Medical News

**Published:** 1852-02

**Authors:** 


### MEDICAL NEWS.

Homœopathy.—A full meeting of the Edinburgh Medico-Chirurgicnl Society was held on the 19th November, no less than sixty-four members being present. After the transaction of routine business, the following interesting circumstances occurred :—

Professor Syme, in moving "That the public profession of homœopathy shall be held to disqualify for being admitted or remaining a member of the Medico-Chirurgical Society," said,—that in addressing the Society on this occasion, he considered it to be quite unnecessary to enter into a formal refutation of the principles of homœopathy, but before proceeding further, he would exculpate himself from the charge of inconsistency brought against him by Dr. Henderson, to the effect that he had himself countenanced homœopathy in two instances. This charge appeared at the time in the various medical periodicals. Now, regarding this inconsistency as tantamount to a practical falsehood, he (Mr. Syme) took the present opportunity of exhibiting the falsities of the accusation. The cases to which Dr. Henderson alludes are two in number. The fact is, there was a young man who had been under the care of Dr. Nimmo, who had been placed under his (Mr. Syme's) care. Finding that he had been attended by Dr. Henderson, Mr. Syme requested a meeting, not for the purpose of consultation, but to arrange for placing the medical treatment under the hands of another physician—Dr. John Taylor—as Mr. Syme felt that he could not co-operate with Dr. Henderson. In the second case, he met Dr. Henderson, being under no pledge not to do so. This is the whole extent of his countenance of homœopathy. Mr. Syme next stated what he conceived to be the duty of every member of the Profession. As an individual, he had long refused to adopt homœopathy, because he regarded it as mischievous folly. As a member of a licensing board, he would not refuse any candidate who complied with the regulations of the University. If such an one were base enough to disguise his real sentiments in regard to the practice of physic, the disgrace would rest with him and not with the Board. The duty of a Society like the present was, he said, clear. It was a voluntary Association for upholding sound principles of practice, and for elevating professional character. If, therefore, a member departed from the principles of the Society, and placed himself in opposition to them, he should be requested to withdraw from their body; or, if seeking admission, he should be excluded. He trusted the motion would be unanimously adopted.

Professor Simpsòn seconded Mr. Syme's motion, and alike also defended himself from the charge of meeting homœopaths in consultation, which emanated from the same quarter. Dr. Henderson affirmed that Dr. Simpson had met him in consultation on some cases; Dr. S., therefore, called upon Dr. Henderson to ask what these cases were. It so happened that Dr. Simpson had anxiously attended Dr. Henderson's own wife; but she, with her husband's sanction, was treated on the ordinary principles of scientific medicine, and not homœopathy. In doing this, no one would accuse Dr. Simpson of countenancing homœopathy. Dr. Henderson, however, mentioned two cases,—one of uterine disease, and the other of disease of the labium. Dr. Henderson had, it is true, previously attended them, and had asked Dr. Simpson to take charge of them, but he did not attend them with, or for, Dr. Henderson. In one other case he certainly did meet Dr. Henderson at the bed-side, but this was a case involving an operation, and not internal treatment; but even in doing this much, Professor Simpson was now convinced he acted wrongly; he had erred in going thus far. However, even if Dr. Henderson's statements had been true to a far greater extent, it only proved, that hitherto the Profession had been over-indulgent to him and his heresy, believing, as his friends did, that the delusion would soon subside. But, because they had been over-tolerant, it was no reason why they should continue so. It now became the duty of the Society to make their stand, feeling that every proper consideration for themselves and the noble science they cultivate, calls imperatively for a complete casting off of homœopathic practitioners, as abettors of delusions and errors. Dr. Simpson continued to draw a parallel between these impostors and the brothers in another profession—Joe Smith and the Mormons. These two heresies, the homœopathic and the Mormonite, had in fact many points in common, and even both equally absurd. Some homœopaths profess Hahnemann to have been despised, [Witness the ravings of the clerical witling quoted by Dr. Cormack.—Ed. P. J.] as the Mormonites do of Joe Smith. It is true, we have no standard of faith whereby to test medical opinion, but we have the standard of common sense. Judged by this, Hahnemann's dogmas are a tissue of the strangest contradictions and the wildest absurdities. If a grown up man were seriously to say that two and two make five, he would not be considered sane, as he defies the dictates of common sense. When other grown up men tell the world, that they can cure this or that disease by the billionth or decillionth of a grain of a drug, they express an opinion more palpably absurd than that of him who says two and two make five. If men would reflect what a billion or a decillion really is, they would not be so childishly credulous. There is no poison so strong that a billionth of a grain would in the least affect a fly, much less a human being. These people tell us, that division of their drugs invests them with "tremendous" power, but one of these "tremendous" billionths will not affect the frailest insect. For what is a billionth of a grain? Why, if a grain of sulphur were divided into billionths, and our first parent, Adam, had, when living, (6,000 years ago,) swallowed one such billionth every second until the present time, supposing he had lived so long, he would not yet have swallowed half the grain. He must work on at the rate of sixty billionths minute for 24,000 years to come to get through the entire grain. Yet, forsooth, these wiseacres believe that a few of these billionths will cure an attack of jaundice. Appealing to common sense, should not such men be requested to withdraw from scientific societies like the present? However, a billionth of a grain is a large dose; some give the tenth dilution or the decillionth of a grain. Little do they reckon what a decillion is. Here is its notation:—1,000,000,000,000,000,000,000,000, 000,000,000,000,000,000,000,000,000,000,000. Now, the world contains nine hundred millions of human beings. If all these nine hundred had lived from the commencement of the world, and each had swallowed every second of their existence a decillionth of a grain, they would not yet have finished the grain. And, say the homœopaths, a few of these decillionth globules of belladonna will cure scarlet fever. One remark of Mr. Syme reminded Dr. Simpson of a curious fact in the early history of homœopathy in Edinburgh, proving on the one hand how far imagination will go, on the other hand, that all homœopathic globules are alike, or rather alike inactive. Some eight years ago Dr. Simpson received a present of a box of homœopathic medicines from an old schoolfellow, who had set up as a homœopathic druggist. During the time it was in Dr. Simpson's possession it was given as a plaything to his son, then a child. The boy amused himself by uncorking the bottles, emptying their contents into a general heap, and then refilling them promiscuously. The effect of this was a complete compounding of the globules of different kinds, by mixing them together. It soon happened that a professional brother calling at Dr. Simpson's took a fancy to the box and carried it off. Many weeks after, the new proprietor of the box met Dr. Simpson, and told him he had been trying homœopathy with the contents of his box, and that he had accomplished wonderful cures. Dr. Simpson enjoyed the joke, and said nothing about the box, until, finding his friend had got deep into the homœopathic mine, and actually published a list of cases, he at length told him of the elaborate mixture the globules had undergone. This friend is Dr. Henderson !!! In conclusion, Dr. Simpson alluded to those impostors, who, pretending to be homœopathists, prescribed ordinary doses in the guise of globules, and practised either way, as best suited their own pockets and the caprices of the patient. These, he argued, should be expelled from the Society.

At the conclusion of this instructive as well as important meeting, the globulists, Drs. Rutherford, Russell, and James Russell, had the good sense to anticipate expulsion by resigning their seats as members of the Society.—*Provincial Medical and Surgical Journal.*

OBITUARY.—We are pained to record the death of Dr. JAMES CAMERON, one of the oldest and most reputable practitioners of our city. There are few among us who have acquired a more extensive practice, or enjoyed a higher degree of the confidence and affections of his patients, which he retained during a long life. Dr. C. was a native of Scotland, a graduate of Glasgow, and a worthy member of the New York Academy of Medicine. He was deservedly respected, and his loss will be deplored, for he was an honest man.—*N. Y. Med. Gaz.*

Dr. SYDNEY A. DOANE, Health Officer, at Quarantine, Staten Island, New York, died of ship-fever, on the 27th of January.

Dr. JAMES ARTHUR, one of the most distinguished medical officers in the British service, died lately in Cheltenham, England, at the age of seventy.

THE ROYAL SOCIETY OF ENGLAND.—The anniversary meeting was held on Monday, the Earl of Rosse, president, in the chair. His lordship delivered his annual address, after which the Copley medal was presented to Professor Owen for his important discoveries in comparative anatomy and palæontology, published in the *Philosophical Transactions;* one of the royal medals to the Earl of Rosse for his observations on the nebulæ and the second royal medal to Mr. G. Newport for his paper on the Impregnation of the Ovum. The Society then proceeded to the election of council and officers for the ensuing year, and the following noblemen and gentlemen were elected:—*President,* the Earl of Rosse, K. P., M. A. *Treasurer,* Lieutenant Colonel Edward Sabine, R. A. *Secretaries,* Mr. Samuel Hunter Christie, M. A., and Mr. Thomas Bell. *Foreign Secretary,* Captain W. H. Smith, R. N. *Other Members of the Council,* Mr. William Bowman, Mr. Benjamin Collins Brodie, Mr. Charles Brooke, the Rev. Professor Challis, M. A., William Clarke, M. D., Charles Bridle Daubeny, M. D., Sir P. de Malpas Grey Egerton, Bart., the very

Rev. the Dean of Ely, Mr. J. P. Gassiot, Marshall Hall, M. D., Sir John Frederick W. Herschel, Bart., Professor W. Hallows Miller, M. A., Lieutenant Colonel Portlock, R. E., Mr. Edward Solly, Mr. William Spence, Nathaniel Wallich, M. D.—*London Lancet.*

---

The London Epidemiological Society.—On the formation of this Society, application was made by the President, Dr. Babington, to various public bodies for their assistance and co-operation; the fruit of this judicious step is now being realized. The Committee on small-pox and vaccination have deputed two of their members to examine the returns annually made by the medical officers appointed by the various boards of guardians according to the requirement of the Act of Parliament, passed in 1840, "to extend the practice of vaccination." Through the courtesy of the Poor-law Board, every facility for the full examination of these returns has been afforded; and we are informed by the honorary secretaries of the Society, that a large body of the most valuable and elucidative information has by these means been obtained by the deputation. Our readers will be enabled to judge of the importance of these official documents, when we state that they show the total number of persons vaccinated yearly by the public vaccinators in each district of the 631 poor-law unions in England and Wales; giving in separate columns those who are under one year, and those above one year of age, and likewise distinguishing in most instances the successful from the unsuccessful vaccinations, under and above one year. As the number of births in each union is also appended, a certain basis is afforded for determining the exact progress of vaccination in every part of England and Wales, so far as the gratuitous system is concerned. The results are now under the consideration of the gentlemen who have gone over the returns; and although it would be premature at the present moment to state the conclusions to which they point, we may affirm that they promise to constitute one of the most trustworthy contributions hitherto made in this country to the statistics of vaccination.

---

Arsenical Poisoning.—A very interesting case has recently come before the French Tribunals, in which the question arose, as to the possible contamination of a corpse buried in an arseniferous soil. M. Barse, who was ultimately consulted, appears to have shown more than ordinary toxicological acumen. (*Union Médicale,* Sept. 1851.)

A man and his wife were accused, the one of poisoning his former wife, the other her former husband. Both coffins, buried in different cemeteries, were exhumed. The female was in good preservation, the coffin being intact; the male less so, the coffin being much decayed, and earth being found within it. Arsenic was found in both bodies, but the surrounding earth in both cemeteries was found to contain arsenic also. The chemists were puzzled, but, from the fact that in the coffin of the female no arsenic could be detected in the cerements or other matters interposed between the corpse and the earth, they concluded that it was a case of poisoning.

M. Barse being appealed to, decided that this was the correct opinion, as the native arsenic in the soil was always mixed with iron, cobalt, or antimony, and could not be detected by ordinary reagents. Its solubility would also prevent its reaching the bodies by filtration. To confirm this view, he likewise examined other bodies buried in the vicinity of the two in question, but no arsenic could be detected. He further searched the chamber in which the man died, and finding splashes on the walls and on the floors, which indicated the vomitings of the patient, had the spots scraped carefully, as also other parts of the room, distant from the bed. The former matters contained arsenic, the latter none. The parties, on this, were found guilty.

Death of Priessnitz.—Priessnitz, the founder of hydropathy, died at Graefenberg on the 26th of November, at the age of fifty-two. In the morning of that day Priessnitz was up and stirring at an early hour, but complained of the cold, and had wood brought in to make a large fire. His friends had for some time believed him to be suffering from dropsy of the chest, and at their earnest entreaty, he consented to take a little medicine, exclaiming all the while, "It is of no use." He would see no physician, but remained to the last true to his faith. About 4 o'clock in the afternoon of the 26th, he asked to be carried to bed, and upon being laid down he expired.

The French Revolution.—On Friday, the 5th inst., Dr. Puriss, of the Rue-de-la-Paix, was mortally wounded in the Rue Faubourg Poissoniere, during his professional visits. He received two musket shots, and died in two hours afterwards. In the same room where he was lying, there were also thirty-five dead bodies.

The London Medical Times and London Medical Gazette have been incorporated, and the joint publication has been issued from the commencement of the current year, under the title of the Medical Times and Gazette.

ANNUAL PUBLIC MEETING OF THE ACADEMY OF MEDICINE OF PARIS. —The Academy held its annual meeting on December the 16th, 1851. A general report was read as usual on the prizes awarded by the Academy in 1851. The subjects of the prizes for 1852 and 1853, were then made known, and M. Dubois (d'Amiens) read a panegyric discourse on Hallé. The prize of the Academy on White Swelling, was awarded to M. Richet, well known for his successful labors on the subject. Madame de Civrieux's prize on Convulsions was not awarded to any of the competitors. A prize on Melancholia only produced an encouragement. Among the subjects for 1853, we notice the following:—" Is there any Paraplegia independent of Myelitis?" The subject of Portal's prize is, "The Anatomy and Physiology of the various kinds of Goitre." Madame de Civrieux's, " The History of Tetanus." Dr. Capuron's, " On the Physiological and Pathological Circumstances of the Puerperal State." The Argenteuil prize, on " The Therapeutics of Stricture," has now accumulated to 480*l.*; it is an hexennial prize, and was not awarded from 1838 to 1844. The Academy prize for 1852 is, " On Ergot of Rye as regards Physiology, Midwifery, and Hygiene." Portal's for 1852, "On the Pathological Anatomy of the Inflammation of Bone;" and Madame de Civrieux's, for the same year, " The Ætiology of Epilepsy: inquire for the indications which the study of its causes may furnish in regard to the preventive or curative treatment of the disease."—*Lon. Lancet.*

